# Integrated Transcriptomic and Metabolomic Insights into “I See You” (ISY) Defensive Behavior in *Apis cerana* Against *Vespa velutina*

**DOI:** 10.3390/insects16101047

**Published:** 2025-10-13

**Authors:** Yijie Chen, Xueling Xu, Yingjiao Li, Ning Ji, Yiwei Ruan, Mei Yang, Hongji Huang, Liulin Yang, Xiaoyu Cao, Jianghong Li

**Affiliations:** 1 State Key Laboratory of Physical Chemistry of Solid Surfaces, Key Laboratory of Chemical Biology of Fujian Province, Collaborative Innovation Center of Chemistry for Energy Materials (iChEM), College of Chemistry and Chemical Engineering, Xiamen University, Xiamen 361005, China; 2 College of Bee Science and Biomedicine, Fujian Agriculture and Forestry University, Fuzhou 350002, China; 3 Fujian Honey Bee Biology Observation Station, Ministry of Agriculture and Rural Affairs, Fuzhou 350002, China

**Keywords:** honeybees, *Apis cerana*, transcriptome analysis, metabolism, defensive behavior

## Abstract

*Vespa velutina* (*V. velutina*) is a globally widespread predator of honeybees, posing a serious threat to them. *Apis cerana* (*A. cerana*) has acquired the ability to partially thwart the predation of the wasps, but the molecular mechanism by which it responds to *V. velutina* attacks is unknown. Here, both transcriptomic and metabolomic analyses were integrated to decipher what happened in the brain tissues of *A. cerana* exposed to *V. velutina* attacks. Transcriptomic analysis revealed 296 differentially expressed genes (DEGs) that were significantly enriched in neural signaling pathways (particularly serotonin and dopamine transmission), the cAMP signaling pathway, and energy metabolism. Metabolomic results showed that *V. velutina* attacks affected 38 metabolic pathways involving 86 differentially expressed metabolites (DEMs), primarily including amino acids, nucleotides, and carbohydrates. Several metabolites, such as isocitrate, xanthine, and adenosine, were upregulated in response to the hornet presence. Combined pathway analysis indicated an upregulation of the purine and tyrosine metabolism pathways, highlighting their critical role in *A. cerana* against invasive hornets. The results of this study will help to understand the molecular mechanisms of *A. cerana* in response to *V. velutina* attacks and provide valuable insights for enhancing collective defensive behavior against *V. velutina* in honeybees.

## Simple Summary

Hornets are major predators of honey bees. When a hornet approaches the nest, guard bees of *Apis cerana* perform an “I See You” (ISY) display. To explore what supports this rapid, coordinated display, we measured changes in brain gene activity and small molecules in bees performing ISY versus routine activity. ISY was associated with shifts in key brain messengers (including serotonin and dopamine) and with increases in molecules that supply energy. Together, these changes may help bees send signals faster and act in sync, sustaining the defensive wave. Understanding the biology behind shimmering offers a simple explanation for how colonies deter hornets and may provide practical clues.

## 1. Introduction 

*Vespa velutina* (*V. velutina*), a wasp endemic to Southeast Asia, has become a significant stressor for honeybees [1]. *V. velutina*, which hawks foraging honeybees at their colonies, can reduce the strength of honeybee colonies and even cause their subsequent collapse. Notably, although many *Apis cerana* (*A. cerana*) colonies are preyed upon by hornets, the losses suffered by *Apis mellifera* (*A. mellifera*) colonies are even more severe [2]. This primarily leads to a reduction in honeybee populations in the environment, as well as causing economic damage to the beekeeping industry [3]. Therefore, *V. velutina* poses a severe threat to beneficial insects like honeybees, leading to declines in ecosystem pollination services and economic losses.

On the other hand, honeybees have developed defense techniques in response to their predators [4]. The honeybee *A. mellifera* exhibits some defensive capability against the native European hornet, *Vespa crabro*, but is unable to adopt an effective defense against *V. velutina* [5]. The aggressive defensive behavior of *A. cerana* is more efficient than that of *A. mellifera*. While both species employ predator suffocation and heat generation (a behavior known as balling), *A. cerana* elevates the internal temperature of the ball faster and higher than *A. mellifera*, and involves a larger number of bees in the ball [6,7]. *A. cerana* also use wing shimmering to create a visual interference mechanism, which is absent in the behavioral patterns of *A. mellifera* [8].

Therefore, the evolved sophisticated colony-level defense is the strategy used by honeybees (e.g., *A. cerana*) to cope with sympatric wasps [9,10,11]. *A. cerana* utilizes wing shimmering as a visual pattern disruption in the presence of *V. velutina*, serving as the innate “I See You” (ISY) signal [12,13]. When hornets approach the nest entrance, *A. cerana* bees vigorously shake their abdomens from side to side in unison and rhythmically fan their wings to produce shimmering waves [14,15]. However, *A. cerana* defensive behaviors against this wasp involve a network of genes, signaling pathways, and metabolic processes rather than a simple linear response. Consequently, it is necessary to investigate the alterations in genes and metabolites in *A. cerana* to elucidate its responses to *V. velutina* attacks.

Here, we performed a comparative metabolomic and transcriptomic analysis of 15-day-old *A. cerana* workers during normal activity and while producing the shimmering signal, aiming to detect dynamic changes and to elucidate the metabolism-related adaptive mechanisms involved in their response to *V. velutina* attacks. The purposes of this study were to (a) identify differentially expressed genes (DEGs) through transcriptomic analysis; (b) determine differentially expressed metabolites (DEMs) and their important metabolic pathways; and (c) map a connection network based on the associations between metabolites and genes in *A. cerana* against predators. Therefore, the current evidence highlights the roles of multiple metabolites and gene regulatory networks in the shimmering behavior exhibited by *A. cerana* workers in the presence of the predator *V. velutina*. These results provide a valuable reference for identifying key genes and metabolites involved in the aggressive defensive behaviors of guard bees.

## 2. Materials and Methods

### 2.1. Honeybees

The Asian hive bee (*A. cerana*) is often attacked by hornets (*V. velutina*). In the co-evolution of these two species, *A. cerana* shows a specific defensive behavior, known as shimmering behavior, in the presence of *V. velutina* [12]. In this study, we used the Chinese honeybee (*A. cerana*), a subspecies of the Asian honeybee native to China that has long been naturally exposed to *V. velutina* in the local environment. Asian hive bees *A. cerana* and *V. velutina* were obtained from an apiary at the College of Bee Science and Biomedicine, Fujian Agriculture and Forestry University (Fuzhou, China), from October to November 2024.

Worker honeybees exhibit an age-dependent division of labor and actively engage in guarding and defensive behaviors, including ISY signaling [16]. Therefore, the following experiment was undertaken to collect 15-day-old bees, which are known to exhibit collective defense behaviors [17]. Frames with old pupae (1 day before emergence) were randomly obtained from the healthy colonies. The combs were then incubated for 24 h at 34 °C with 75% relative humidity in darkness. Newly emerged bees were marked with paint on their thoraxes and returned to their original colonies. After 15 days, marked bees that were engaged in routine colony activities and not exposed to external stimuli were collected as the control group (CK). The ISY signaling behavior was induced by presenting a tethered *V. velutina* with its stinger removed. The hornet was fixed to the end of a stick and positioned 10 cm in front of *A. cerana* hive entrances. Marked workers that gathered at the nest entrance and participated in shimmering behavior in response to the hornet were collected using long tweezers. These individuals were assigned to the ISY group (ISY). All collected bees were immediately frozen in liquid nitrogen within seconds of capture.

For each group (CK and ISY), we sampled three independent colonies from the same apiary (one colony = one biological replicate; n = 3 per group). In each replicate, twenty 15-day-old workers were pooled to generate one sample. The samples were frozen in liquid nitrogen and subsequently stored at −80 °C for further research.

### 2.2. RNA Extraction, Library Construction, and Sequencing

Brain tissues from each sample were used to extract total RNA with TRIzol reagent (Invitrogen, Carlsbad, CA, USA) according to the manufacturer’s protocol. The quantity and integrity of RNA were assessed using an ND-2000 spectrophotometer (NanoDrop Technologies, Wilmington, DE, USA) and agarose gel electrophoresis, respectively. RNA-Seq was performed by Biozeron Co., Ltd. (Shanghai, China). Then, 1 µg of RNA from each sample was used as input material for the RNA sample preparation. cDNA libraries were constructed with the TruSeq™ RNA Sample Preparation Kit (Illumina, San Diego, CA, USA). The final libraries were quantified using TBS380 and sequenced on the Illumina NovaSeq 6000 platform (Illumina, San Diego, CA, USA).

After filtering the raw data and checking the sequencing error rate and GC content distribution, clean reads were obtained for downstream analyses. The mapped data were then generated by sequence alignment with the *A. cerana* reference genome (GCF_029169275.1, NCBI) (https://www.ncbi.nlm.nih.gov/datasets/genome/GCF_029169275.1/ (accessed on 6 January 2025)). Fragments Per Kilobase of Transcript per Million fragments mapped (FPKM) values were used to indicate gene expression levels. The DEG screening criteria were a |log 2FC| ≥ 1 and an FDR < 0.05. The screened DEGs were subjected to Kyoto Encyclopedia of Genes and Genomes (KEGG) and Gene Ontology (GO) enrichment analyses. Genes were regarded as significantly enriched in GO terms and KEGG pathways when *p* < 0.05. We used the edgeR package version 3.6.3 (http://www.bioconductor.org/packages/release/bioc/html/edgeR.html (accessed on 6 January 2025)) for statistical analysis and visualization.

### 2.3. Fluorescent Real-Time Quantitative PCR (qRT-PCR)

To validate transcriptome findings, 6 DEGs were chosen for quantitative real-time PCR (qRT-PCR) analysis, with *β-actin* serving as the internal reference gene. The PrimeScript™ RT Reagent Kit (Takara Bio, San Jose, CA, USA) was employed to synthesize cDNA. Reactions were performed on an ABI QuantStudio 6 Flex System (Thermo Fisher Scientific, Waltham, MA, USA). qRT-PCR was conducted with SYBR Green Master Mix (Yeasen Biotech Co., Ltd., Shanghai, China) under the following conditions: 95 °C for 3 min, followed by 45 cycles of 95 °C for 15 s, 60 °C for 20 s, and 72 °C for 20 s. Each sample was repeated three times, and the 2^−ΔΔCt^ method was used to analyze quantitative data. The primer sequences are listed in Supplementary Table S1.

### 2.4. Data Analysis

Brain tissues from *A. cerana* from each group were prepared for non-targeted metabolomics analysis. For sample extraction, 100 mg of tissue from each sample was ground individually in liquid nitrogen. The resulting homogenate was resuspended in prechilled 80% methanol with 0.1% formic acid and incubated on ice for 5 min. The mixture was centrifuged at 4 °C for 5 min at 15,000 rpm. A portion of the supernatant was diluted with mass spectrometry water to reach a final methanol concentration of 53%. The diluted solution was then transferred to a new EP tube and centrifuged at 15,000× *g* at 4 °C for 10 min. Finally, the supernatant was collected into an injection vial for analysis. Each treatment group included three biological replicates.

Metabolite profiling was performed using a non-targeted metabolomic approach by Biozeron Co., Ltd. (Shanghai, China). The sample extracts were examined on a Vanquish UHPLC system (Thermo Fisher, Dreieich, Germany) coupled to an Orbitrap Q Exactive™ HF mass spectrometer (Thermo Fisher, Dreieich, Germany). Raw data processing was carried out using Compound Discoverer 3.1 software (Thermo Fisher Scientific), which performed automated peak alignment, feature detection, and compound quantification. The peak data were aligned to the mzCloud (https://www.mzcloud.org/), mzVault, and MassList databases within the software for annotation.

The orthogonal partial least squares discriminant analysis (OPLS-DA) model was performed to generate values of the variable importance in projection (VIP). DEMs between groups were determined using the cutoff criteria VIP > 1 and fold change (FC) ≥ 2 or FC ≤ 0.5. Identified DEMs were annotated using the KEGG database. Pathways with *p* < 0.05 were considered to be significantly enriched.

### 2.5. Integrated Metabolomic and Transcriptomic Analysis

Correlation analyses between the genes and metabolites in each group were performed. Pearson correlation coefficients were computed using R software (v3.6.3). In addition, DEGs and DEMs from the same group were simultaneously mapped to KEGG pathway charts and visualized. This process was carried out based on the outcomes of differential metabolite analysis and transcriptomic analysis, respectively.

### 2.6. Statistical Analysis

All experiments in this study were carried out with three biological replicates. The data were analyzed using GraphPad Prism 7.0 (Boston, MA, USA). The results were presented as mean ± standard deviation (SD). * *p* < 0.05 indicates a significant difference.

## 3. Results

### 3.1. Transcriptome Sequencing Results and Quality Analysis

To elucidate the molecular regulatory mechanisms of intensified self-defensive behavior (ISY) in honeybees, we performed RNA-Seq on 15-day-old worker bees. Six samples were analyzed via transcriptome sequencing. After removing low-quality reads and connector contamination, we obtained a total of 33.8 Gb of clean data, with average Q20 and Q30 scores of 98.85% and 96.44% (Table S2). The clean reads were compared and annotated. High mapping rates of clean reads to the *A. cerana* reference genome (96.23–97.98%) confirmed successful library preparation and sequencing accuracy (Table S2). These results indicated that the sequencing data were of sufficient quality for subsequent analysis.

**Figure 1:**
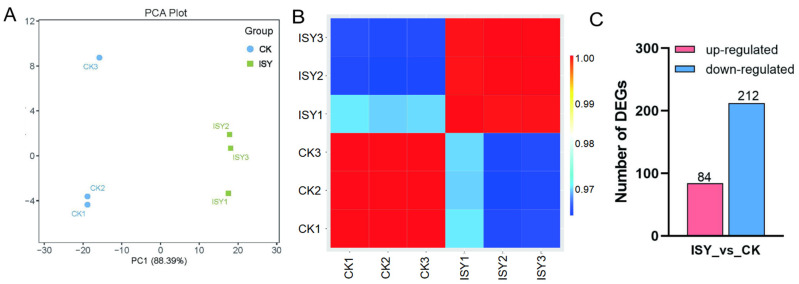
Transcriptomic alterations in *A. cerana* workers in the presence of *V. velutina*. (**A**) Principal component analysis (PCA) of the samples. (**B**) Heatmap of correlation between ISY and CK groups. The false color scale is displayed on the right side of the image. (**C**) Number of differentially expressed genes (DEGs) between the ISY and CK groups.

First, a principal component analysis (PCA) of the gene expression data revealed that the first two principal components clearly distinguished between ISY and CK samples (Figure 1A). Consistent with the PCA results, a correlation analysis revealed that the three biological replicates within each treatment were highly similar, demonstrating the reliability and reproducibility of the data (Figure 1B). The DEGs were then analyzed to further explore the transcriptional changes in *A. cerana* workers during a *V. velutina* attack (Table S3). A total of 296 DEGs (84 upregulated and 212 downregulated) were found in the ISY group compared with the CK group (Figure 1C), using a threshold of FDR < 0.05 and |log2FC| > 1. The top 10 upregulated and downregulated DEGs are presented in Table S4.

### 3.2. Functional Enrichment Analysis of DEGs

**Figure 2:**
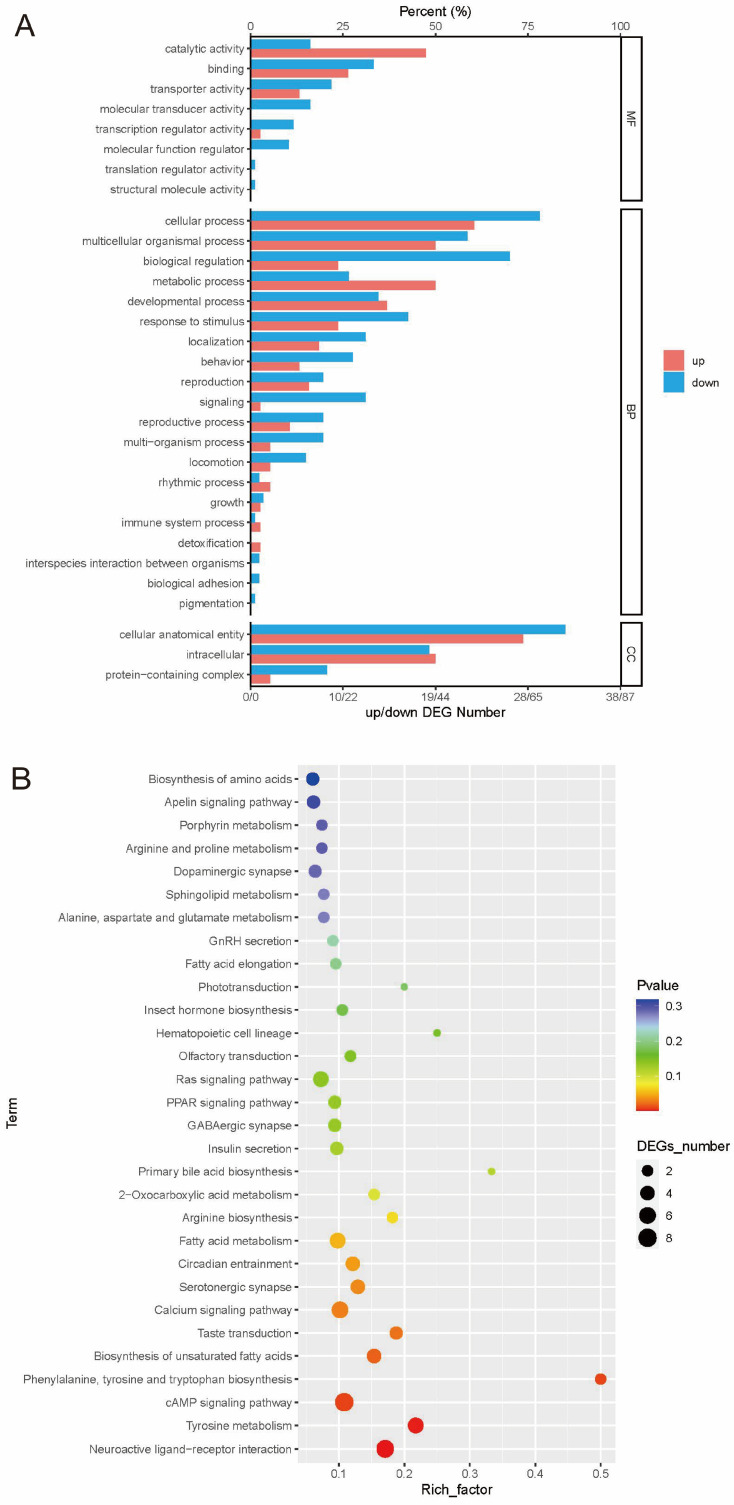
Enrichment analysis of differentially expressed genes (DEGs) in ISY and CK groups. (**A**) GO enrichment analysis. (**B**) KEGG pathway enrichment analysis.

To investigate the biological function of these DEGs, GO and KEGG analyses were performed. In the GO analysis, all DEGs were annotated using the GO database and then grouped into three categories: Biological Process (BP), Cellular Component (CC), and Molecular Function (MF). Compared with the CK group, the greatest number of DEGs in the ISY group were found in the BP category, and were enriched in multicellular organismal processes (GO:0032501), response to stimulus (GO:0050896), and cellular response to stimulus (GO:0051716). Within the CC category, the DEGs were mainly enriched in the membrane (GO:0016020) and integral component of membrane (GO:0016021). For the MF category, the DEGs were mainly enriched in transporter activity (GO:0007154) and transmembrane transporter activity (GO:0022857) (Figure 2A, Table S5).

Additionally, KEGG analysis was performed on all DEGs, and a total of 118 pathways were enriched. The top 30 pathways are shown in Figure 2B. Compared to the CK group, the KEGG results showed that *A. cerana* mainly thwart the predations of the wasps through neuroactive ligand–receptor interaction (ko04080), tyrosine metabolism (ko00350), and the cAMP signaling pathway (ko04024) (Figure 2B, Table S6).

### 3.3. Validation of Transcriptomic Data by Quantitative Real-Time PCR (qRT-PCR)

**Figure 3:**
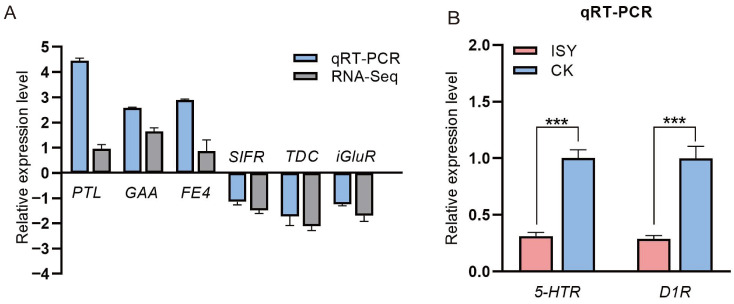
qRT-PCR analysis of differentially expressed genes (DEGs). (**A**) Key gene expression levels were determined using qRT-PCR. (**B**) Changes in *5-hydroxytryptamine receptor* (*5-HTR*) and *dopamine receptor 1* (*D1R*) genes. *** indicates significant difference (*p* < 0.001, *t*-test).

To validate the RNA-Seq results, we performed qRT-PCR on a subset of differentially expressed genes (DEGs), including the upregulated genes *PTL*, *GAA*, and *FE4*, and the downregulated genes *SIFR*, *TDA*, and *iGluR*. These genes were consistent with the expression pattern observed in the transcriptomic analysis, which confirmed the reliability of the transcriptome data (Figure 3A). Moreover, we found that the expression levels of candidate genes related to biogenic amines, including the *5-hydroxytryptamine receptor* (*5-HTR*) gene and the *dopamine receptor 1* (*D1R*) gene, were markedly reduced in the ISY group compared to the CK group (Figure 3B).

### 3.4. Metabolomic Analysis

**Figure 4:**
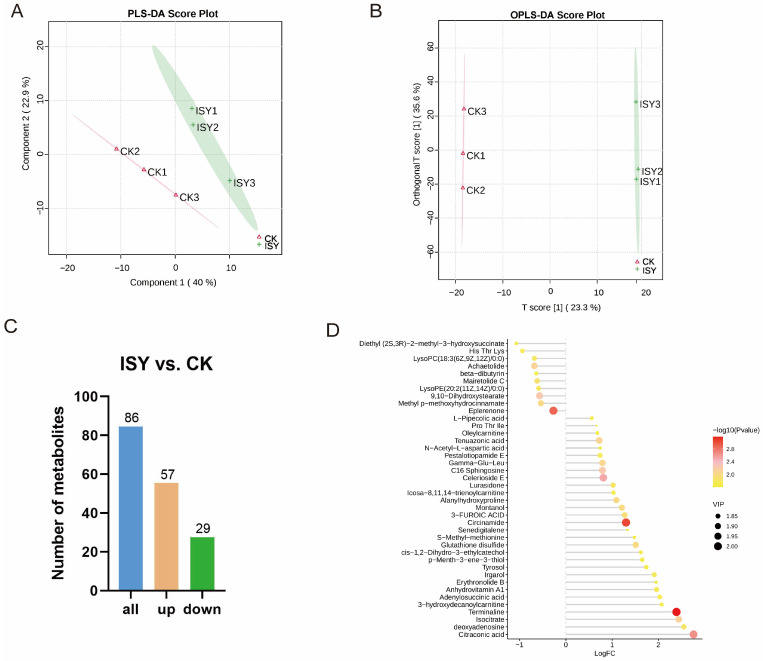
Changes in metabolites in ISY and CK groups. (**A**) Analysis of differentially expressed metabolites (DEMs) based on PLS-DA scores. (**B**) OPLS-DA analysis. (**C**) Different metabolite counts. Yellow and green, respectively, indicate significantly upregulated and downregulated metabolites. (**D**) Top 30 DEMs between comparison groups.

A metabolomic analysis of the ISY and CK groups was conducted to comprehensively understand the effect of the *V. velutina* treatment on *A. cerana* workers. Partial least squares discriminant analysis (PLS-DA) and OPLS-DA were applied to examine the clustering patterns of the two groups. Clear separation of metabolites between the ISY and CK groups was observed, and the three biological replicates of each group were tightly grouped, demonstrating the reproducibility and reliability of the experiment (Figure 4A,B).

In total, 86 distinct metabolites were detected, including 30 lipids and lipid-like molecules, 23 organic acids and derivatives, 12 organoheterocyclic compounds, 6 nucleotides and analogues, 6 organic oxygen compounds, 4 benzenoids, 3 phenylpropanoids and polyketides, and 2 organic nitrogen compounds (Table S7). Among them, 57 upregulated and 29 downregulated metabolite profiles were identified in ISY (Figure 4C). The levels of organic acids and nucleotides were higher than those of other metabolites, suggesting that these two metabolites played a crucial role in protecting *A. cerana* against *V. velutina* (Figure 4D, Table S8). Taken together, the data indicated that *A. cerana* workers exhibit different metabolite accumulation patterns when confronted with *V. velutina*, highlighting the role of differential metabolites as conserved stress response metabolites.

**Figure 5:**
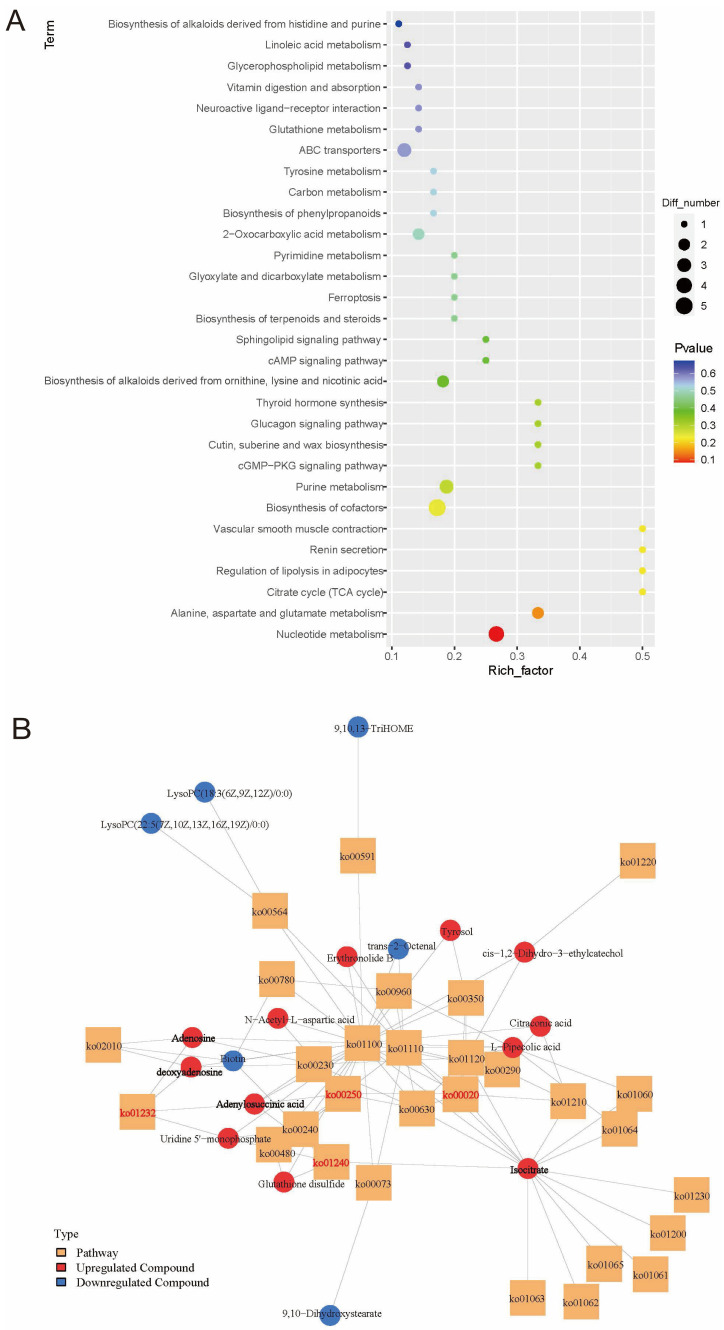
Analysis of metabolic pathways of differentially expressed metabolites (DEMs). (**A**) KEGG enrichment histogram of the 30 pathways containing significantly enriched DEMs. (**B**) Analysis of the regulatory network of DEMs. Each orange box represents a KEGG pathway, and each circular dot indicates a metabolite.

The DEMs were further analyzed using the KEGG database. DEMs were predominantly enriched in metabolic pathways, including those related to nucleotide metabolism (ko01232); alanine, aspartate, and glutamate metabolism (ko00250); the citrate cycle (TCA cycle) (ko00020); and the biosynthesis of cofactors (ko01240) between the ISY group and the CK group (Figure 5A). The DEMs enriched in these four significant metabolic pathways included uridine 5′-monophosphate, adenosine, deoxyadenosine, adenylosuccinic acid, and isocitrate (Figure 5B). These enriched pathways supported robust metabolic adjustments underlying the intensified neural and physiological responses observed during ISY behavior.

### 3.5. Integrated Analysis of Metabolomic and Transcriptomic Data

**Figure 6:**
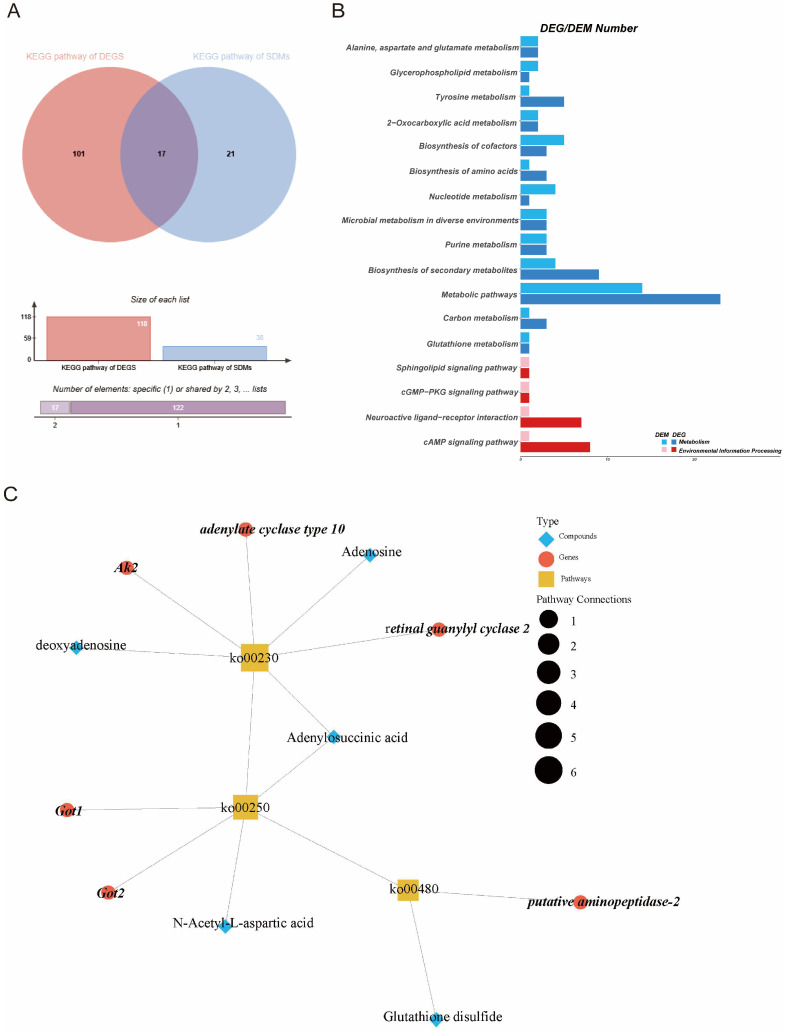
Correlation analysis of differentially expressed genes (DEGs) and differentially expressed metabolites (DEMs). (**A**) Venn diagram for KEGG of DEGs and DEMs. (**B**) Enrichment pathways of DEGs and DEMs in ISY vs. CK comparison. (**C**) The analysis of transcriptome and metabolome was integrated to construct an interaction network. Red circles, genes; blue diamonds, differential metabolites; yellow squares, metabolic pathways.

The KEGG analysis of DEGs and DEMs showed that there were 17 co-enriched KEGG pathways, mainly metabolic pathways (ko01100), tyrosine metabolism (ko00350), and purine metabolism (ko00230) (Figure 6A,B). These pathways were primarily associated with nucleotide metabolism and amino acid metabolism.

The network analysis revealed that the differential metabolites and DEGs were mainly concentrated in purine metabolism (ko00230); alanine, aspartate, and glutamate metabolism (ko00250); and glutathione metabolism (ko00480) (Figure 6C). A further Pearson correlation analysis (|r| ≥ 0.9) explored potential regulatory networks between the DEGs and DMs, revealing significant correlations between 37 metabolites and 38 genes (Table S9). The results showed that numerous genes in the energy supply process were associated positively with metabolites in the ISY group compared to the CK group. Notably, 9,10-Dihydroxystearate showed strong correlation with *glutamate oxaloacetate transaminase 2 (Got2)*, suggesting the coordinated regulation of energy metabolism and neuronal signaling (Table S9). These results show that the metabolites within the pathway are directly or indirectly influenced by these genes.

## 4. Discussion

*A. cerana* exhibits a specific defensive behavior, known as “ISY,” which is used to defend against the hornet *V. velutina* [12]. Guard bees recruit a large number of colony members and display shimmering behavior to prevent *V. velutina* from attacking while hovering at the nest entrance [18]. In order to better understand the molecular basis of this behavior, we employed RNA-Seq and metabolomic analyses to measure gene expression and metabolomic profiles in the brains of worker bees during wing shimmering behavior. The comprehensive analysis revealed that there were significant differences in gene and metabolite abundances when comparing the ISY group with the CK group.

We detected a large number of DEGs in the ISY group, suggesting that the presence of hornets (*V. velutina*) stimulates the *A. cerana* to positively activate their defense mechanisms. GO and KEGG enrichment revealed that these DEGs related to *A. cerana* performing synchronized shimmering were predominantly enriched in the neuroactive ligand-receptor interaction, cAMP signaling pathway, and tyrosine metabolism signaling pathway. Further comparison revealed that a suppression was observed in neuroactive ligand–receptor interaction, the cAMP signaling pathway, taste transduction, serotonergic synapse, circadian entrainment, insulin secretion, the GABAergic synapse, the Ras signaling pathway, olfactory transduction, and the dopaminergic synapse in *V. velutina*-exposed bees compared to the controls. An effective innate defensive response against predators requires the brain to encode multiple features of the encounter process, from recognizing hornet stimuli to coordinating motor outputs, thereby facilitating an effective innate defensive response. Notably, the neuroactive ligand–receptor interaction pathway mediates intricate interactions between neurotransmitters and hormones (such as serotonin, dopamine, and GABA) and their respective receptors, playing a crucial role in neural transmission, emotional regulation, motor activities, and guard behaviors [19]. Studies of invertebrate species suggest that biogenic amines are key regulators of aggression [20,21]. Indeed, 5-hydroxytryptamine (5-HT) has been linked to the confrontation or retreat responses in crustaceans [22,23]. Drug-induced increases in 5-HT in the brains of naive flies increases their aggression [24]. Aggression in male *Drosophila* was increased by 5-HT neuron activation, which also restored fighting motivation in loser males [25]. However, we observed that the serotonin signaling pathway in the brains of the ISY group was markedly downregulated compared to the control group. This finding suggests that reduced serotonergic activity may increase bee aggression, which is different from the result of a previous study [26]. Some studies have suggested that 5-HT’s function in modulating aggression depends on the receptor subtypes involved, the type of aggression studied (such as maternal or self-defensive aggression), and the use of animal models [27,28]. Critically, it has been demonstrated that serotonin antagonist upregulates the sting extension reflex in the honeybee [29]. Serotonin avoids excessive responsiveness to irrelevant stimuli, thereby maintaining an appropriate level of responsiveness to harmful stimuli [30]. Therefore, we speculate that reducing the serotonin signals in the brain may enhance the defensive response of bees to noxious stimulations.

The dopamine signal also plays a significant role in regulating defensive behaviors. A study has shown that the levels of brain dopamine were reduced in bees under different stress treatments [31]. The effects of stress on dopamine levels may be related to temporary long-term memory loss. Dopamine D1 receptor (D1R) signaling contributes to the acquisition of contextual fear, and impaired D1R function can affect the formation of fear memory [32]. Dopamine signals that encode both innate and learned sensory valences also mediate memory regulation in the *Drosophila* brain [33]. When exposed to predator threats, dopamine signaling in *Caenorhabditis elegans* modulates its egg-laying behavior, thereby reducing the risk of harm to progeny [34]. In this study, *D1R* gene expression was markedly reduced in the ISY group, whereas proteins such as tyrosine decarboxylase-like related to dopamine and the activity of the dopaminergic synapse pathway were also decreased. This indicates that the threat from wasps has led to the inhibition of the dopamine signaling pathways in the brains of bees. Notably, the taste transduction pathway and olfactory transduction pathways were also downregulated in this group. Similarly, previous studies demonstrated that prolonged exposure to the threat of hornet predators induces defensive clustering behavior in honeybees while reducing brain dopamine levels and impairing sensory and cognitive capacities [35,36]. It has also been proposed that dopamine is essential for attention regulation, helping to prevent responses to irrelevant stimuli [30]. Furthermore, studies demonstrated that biogenic amine signaling modulates neural energetic state in aggression-relevant regions of the honeybee brain [37]. Previous research has established that biogenic amines, including dopamine and serotonin, play a role in driving responses to the predator threat in ants and fruit flies [38,39]. We speculate that during the long-term response to wasp threats, the reduction in serotonergic signaling and dopamine signals may suppress responses to irrelevant, non-predictive stimuli, and allocate energy preferentially to continuous group defense behaviors, thereby enabling efficient behavioral performance.

Furthermore, the cAMP signaling pathway plays a role in synaptic plasticity, learning, and memory processes in invertebrates. The cAMP signaling pathway is associated with the behavioral plasticity during aggressive encounters and the development of the loser effect in *Drosophila* [40]. In honeybees, the cAMP pathway is central to the formation of memory and the integration of sensory inputs [41]. We found that after exposure to the predator *V. velutina*, the cAMP signaling pathway in the transcriptional profile of the bee brain was significantly reduced. Previous research has shown that cAMP signaling is coupled to bee dopamine receptor genes changes [42]. We propose that inhibiting the cAMP signaling pathway might also be an adaptive mechanism for *A. cerana*, temporarily suppressing processes that rely on plasticity, thereby reallocating resources to collective behavioral defenses.

Rapid and effective innate defensive behaviors (such as wing shimmering and abdomen shaking) are critical for *A. cerana* to defend against predators, and this continuous and frequent behavior requires a significant amount of energy [43,44,45]. In our study, DEMs were mostly enriched in amino acid and carbohydrate metabolism pathways, which are responsible for energy synthesis, release, and storage in living organisms. Among these, alanine, aspartate, and glutamate metabolism and the citrate cycle (TCA cycle) are predominantly upregulated. The activation of these pathways not only provides intermediates for energy synthesis, but also directly supplies energy through the oxidation and decomposition of carbohydrates, meeting the high energy consumption demands of the bee brain during defensive behaviors. Consistent with these findings, the expression of genes encoding *alpha-glucosidase* (*GAA*) and *glucose dehydrogenase* (*GDH*), which are involved in sugar metabolism, increased in the ISY group. MP1 neurons through dopamine receptors increase glucose metabolism in the insect brain [46]. The neurons involved in energy metabolism are highly activated in the brains of Japanese honeybee workers fighting against the giant hornet [47]. Therefore, the upregulation of *GAA*- and *GDH*-related genes could promote sugar metabolism in the brains of workers during ISY behavior. Furthermore, aerobic glycolysis can provide bees with more direct energy to cope with the short, high energy consumption situation caused by the hovering wasps near the entrance of the beehive [48,49].

Interestingly, the increased activity of the nucleotide metabolic pathways was markedly upregulated, which was consistent with the genes encoding *adenylate kinase* and *ATP-binding cassette sub-family F member 1*. Based on metabolomics analysis data, it was shown that nucleotide metabolism, especially purine metabolism, was the most perturbed pathway in the fear condition. Xanthine was a key metabolite among those significantly altered in this pathway. It is produced through the oxidation of hypoxanthine, and the intrastriatal injection of hypoxanthine has been shown to impair fear memory processing [50]. The significantly elevated xanthine levels in the ISY group were associated with enhanced memory and olfactory sensitivity in honey bees, thereby increasing their alertness and vigilance [51]. Furthermore, adenosine, an important product of purine metabolism, activates downstream signaling by binding to adenosine receptors, thereby regulating diverse physiological processes like energy metabolism and immune responses [52]. When insects are infected by pathogens, adenosine modulates the redistribution of energy metabolism [53]. Adenosine has been demonstrated to enhance carbohydrate metabolism, thereby increasing glucose levels in the hemolymph of *Spodoptera litura* after adenosine feeding [54,55]. More recently, Lin et al. reported that dietary adenosine promoted energy metabolism and contributed to increasing the movement of worker bees [56]. Of note, adenosine was significantly increased in the ISY group in this work. We can infer from the result that adenosine may serve to provide sufficient energy for the activity of flight muscles and subsequently enhance the rapid wingbeat frequencies of bees to defend against hornets. Adenosine is known to promote carbohydrate metabolism by enhancing glycolysis and the TCA cycle, thereby facilitating ATP production from glucose to meet cellular energy demands [57]. Furthermore, adenosine is associated with aggressive behavior, with the brain energy metabolism of highly aggressive honey bees characterized by increased aerobic glycolysis [48]. Adenosine not only increases energy metabolism in the brain of honey bees, but also enhances memory by promoting memory gene expression, which helps honey bees learn the odors of hornets to defend themselves [36,58].

## 5. Conclusions

In summary, this study employed an integrated transcriptomic and metabolomic approach to investigate the response mechanisms of Asian hive bee workers to the presence of *V. velutina*. Exposure to *V. velutina* triggered rapid physiological changes in *A. cerana* and induced the occurrence of synchronized shimmering behavior. The results showed that the DEGs screened by transcriptome sequencing were primarily associated with neuroactive ligand–receptor interaction, cAMP signaling, and tyrosine metabolism pathways. Metabolomics analyses indicated that metabolic pathways involving amino acids, nucleotides, and carbohydrates were upregulated in the ISY group. Combined analysis indicated that the neural signal transmission and purine metabolism pathways were important in the execution of shimmering behavior by *A. cerana* in response to *V. velutina*. These findings provide a molecular basis underlying the defense strategies employed by *A. cerana* against predators.

## Data Availability

Raw sequencing data have been deposited in the NCBI (PRJNA105148). The other data presented in this study are available on request from the corresponding authors.

## Supplementary Materials

The following supporting information can be downloaded at https://www.mdpi.com/article/10.3390/insects16101047/s1, Table S1. Sequences of primers used for qRT-PCR; Table S2. Summary of RNA sequencing and alignment data for each sample; Table S3. The Fragments Per Kilobase of exon model per Million mapped fragments (FPKM) values of the DEGs; Table S4. The upregulated and downregulated DEGs in compared samples; Table S5. The top 30 GO pathways of DEGs enrichment degree; Table S6. The top 30 KEGG pathways of DEGs enrichment degree; Table S7. Summary of metabolome results; Table S8. Distribution of the DEMs identified in this study among different clusters; Table S9. Correlation between metabolites and DEGs.
